# Is fruit and vegetable intake associated with asthma or chronic rhino-sinusitis in European adults? Results from the Global Allergy and Asthma Network of Excellence (GA^2^LEN) Survey

**DOI:** 10.1186/s13601-016-0140-9

**Published:** 2017-01-27

**Authors:** Vanessa Garcia-Larsen, Rhonda Arthur, James F. Potts, Peter H. Howarth, Matti Ahlström, Tari Haahtela, Carlos Loureiro, Ana Todo Bom, Grzegorz Brożek, Joanna Makowska, Marek L. Kowalski, Trine Thilsing, Thomas Keil, Paolo M. Matricardi, Kjell Torén, Thibaut van Zele, Claus Bachert, Barbara Rymarczyk, Christer Janson, Bertil Forsberg, Ewa Niżankowska-Mogilnicka, Peter G. J. Burney

**Affiliations:** 1 Population Health and Occupational Medicine Group, National Heart and Lung Institute, Imperial College London, London, UK; 2 Department of Nutrition, King’s College London, London, UK; 3 Faculty of Medicine, University of Southampton, London, UK; 4 Skin and Allergy Hospital, Helsinki University Hospital, Southampton, Finland; 5 Immuno-allergology Department, Coimbra University Hospital, Helsinki, Portugal; 6 Department of Epidemiology, College of Medicine, Medical University of Silesia, Katowice, Poland; 7 Department of Immunology, Rheumatology and Allergy, Medical University of Lodz, Coimbra, Poland; 8 Research Unit for Occupational and Environmental Medicine, Institute of Clinical Research, University of Southern Denmark, Coimbra, Denmark; 9 Institute of Social Medicine, Epidemiology and Health Economics, Charité - Universitätsmedizin Berlin, Lodz, Germany; 10 Institute of Clinical Epidemiology and Biometry, Würzburg University, Würzburg, Germany; 11 Deptartment of Pediatrics, Charité – Universitätsmedizin Berlin, Berlin, Germany; 12 Section of Occupational and Environmental Medicine, University of Gothenburg, Odense, Sweden; 13 Upper Airway Research Laboratory, Ghent University, Ghent, Belgium; 14 Division of ENT Diseases, Karolinska Institute, Stockholm, Sweden; 15 Clinical Department of Internal Diseases, Allergology and Clinical Immunology, Medical University of Silesia, Katowice, Poland; 16 Department of Medical Sciences, Respiratory, Allergy and Sleep Research, Uppsala University, Ghent, Sweden; 17 Division of Occupational and Environmental Medicine, Department of Public Health and Clinical Medicine, Umeå University, Chorzów, Sweden; 18 Jagiellonian University School of Medicine, Krakow, Poland; 19 Respiratory Epidemiology, Occupational Medicine and Public Health Group, National Heart and Lung Institute, Imperial College London, Emmanuel Kaye Building, Manresa Road, London, SW3 6LR UK

**Keywords:** Fruits, Vegetables, Asthma, Chronic rhino-sinusitis, Adults, Europe, Meta-analysis, GA^2^LEN

## Abstract

**Background:**

Fruits and vegetables are rich in compounds with proposed antioxidant, anti-allergic and anti-inflammatory properties, which could contribute to reduce the prevalence of asthma and allergic diseases.

**Objective:**

We investigated the association between asthma, and chronic rhino-sinusitis (CRS) with intake of fruits and vegetables in European adults.

**Methods:**

A stratified random sample was drawn from the Global Allergy and Asthma Network of Excellence (GA^2^LEN) screening survey, in which 55,000 adults aged 15–75 answered a questionnaire on respiratory symptoms. Asthma score (derived from self-reported asthma symptoms) and CRS were the outcomes of interest. Dietary intake of 22 subgroups of fruits and vegetables was ascertained using the internationally validated GA^2^LEN Food Frequency Questionnaire. Adjusted associations were examined with negative binomial and multiple regressions. Simes procedure was used to control for multiple testing.

**Results:**

A total of 3206 individuals had valid data on asthma and dietary exposures of interest. 22.8% reported having at least 1 asthma symptom (asthma score ≥1), whilst 19.5% had CRS. After adjustment for potential confounders, asthma score was negatively associated with intake of dried fruits (β-coefficient −2.34; 95% confidence interval [CI] −4.09, −0.59), whilst CRS was statistically negatively associated with total intake of fruits (OR 0.73; 95% CI 0.55, 0.97). Conversely, a positive association was observed between asthma score and *alliums* vegetables (adjusted β-coefficient 0.23; 95% CI 0.06, 0.40). None of these associations remained statistically significant after controlling for multiple testing.

**Conclusion and clinical relevance:**

There was no consistent evidence for an association of asthma or CRS with fruit and vegetable intake in this representative sample of European adults.

## Background

Fruits and vegetables are rich sources of nutrients and compounds with antioxidant, anti-allergic and anti-inflammatory properties, which could modulate the expression of asthma and allergic diseases [1]. A recent systematic review suggested an overall reduced risk of wheeze or self-reported Dr diagnosed asthma in adults and children with higher intakes of fruits and vegetables [2]. Several observational studies in adults have shown a negative association between various asthma prevalence outcomes, and intake of apples [3], citrus fruits [4], tomatoes or leafy vegetables [4]. Smaller studies in asthmatic adults with a dietary pattern mainly comprised of fruits and vegetables have also been shown to have a lower risk of severe asthma [2]. The current evidence on a possible protective effect of fruits and vegetables on allergic diseases is mixed, with some studies showing a negative association between intake of vegetables [5] or food groups that contain them [6] and a lower asthma prevalence, whilst several population-based studies have reported no association between allergic symptoms and fruits or vegetables when measured individually [7, 8] or as part of a dietary pattern [9, 10].

Epidemiological studies use different operational definitions to assess asthma, as well as different instruments to ascertain usual dietary intake. These issues may make it more difficult to ascribe a consistent interpretation on their relationship. The current observational evidence in European adults is inconclusive, with very few multi-national studies examining in some standardised fashion, the association between asthma and diet [10]. Within the Global Allergy and Asthma Network of Excellence (GA^2^LEN), we designed and piloted a single, common, food frequency questionnaire (FFQ) [11], which was used to estimate usual dietary intake of over 3500 adults from 10 European countries participating in the GA^2^LEN Follow-up survey. In this analysis, we investigate the cross-sectional association between asthma and chronic rhino-sinusitis (CRS), with dietary intake of fruits and vegetables in these adults.

## Methods

### The GA^2^LEN study—screening and clinical surveys

The core protocol for the GA^2^LEN survey required 18 European participating centres to identify a random sample of at least 3000 adults aged 15–74 years from an available population-based sampling frame. A stratified random sample was drawn, in which 55,000 adults aged 15–75 answered a questionnaire on respiratory symptoms. The following countries (and cities) were included in this cross-sectional analysis: Belgium (Ghent), Denmark (Odense), Finland (Helsinki), Germany (Duisberg, Brandenburg), The Netherlands (Amsterdam), Poland (Krakow, Lodz, Katowice), Portugal (Coimbra), Sweden (Gothenburg, Stockholm, Umea, Uppsala), and the UK (Southampton, London). In 2008–2009, potential participants were sent a short questionnaire by mail, and at least three attempts were made to elicit a response [12]. The questionnaire collected information on age, gender, smoking and the presence of symptoms of asthma (including age of onset), and CRS. Four sub-samples were selected to define cases and controls: (1) those with self-reported asthma and at least one respiratory symptom reported in the last 12 months (‘asthma’), (2) those having chronic sinusitis (defined following the EP^3^OS criteria, that is, the presence of at least two of the following symptoms for at least 12 weeks in the past year: (i) nasal blockage, (ii) nasal discharge, (iii) facial pain or pressure or (iv) reduction in sense of smell with at least one of the symptoms being nasal blockage or nasal discharge), (3) those who had both ‘asthma’ and ‘chronic sinusitis’, and those who had none of these conditions. [13] Five questions on symptoms in the last 12 months (breathless when wheezing, woken with tightness in chest, shortness of breath while at rest, shortness of breath after exercise, woken by shortness of breath) were used to construct an asthma symptom score on a five-point scale [14].

### Dietary intake

The GA^2^LEN food frequency questionnaire (FFQ) was designed to assess usual dietary intake across countries, using a single, common, and standardised instrument. The FFQ was validated in a random sample of adults from 5 participant centres in GA^2^LEN, namely Finland, Portugal, Germany, Greece, and Poland, each representing a different European Region [11]. All centres adhered to the same standard operational procedure (SOP) to translate the questionnaires and the same procedure was used to translate and standardise all other questionnaires in the GA^2^LEN survey. The GA^2^LEN FFQ has been translated into more than 25 languages for use in several single and multi-national epidemiological studies [15]. To facilitate international food comparisons, the FFQ was organised into 32 sections of food groups [16]. The FFQ collected data on a wide range of foods, including 43 vegetables and 25 fruits (Table 1). Total energy intake (TEI) was calculated using the latest available food composition estimates from the British Food Composition Table [17].

**Table 1:** Fruit and vegetable subgroup classification in the GA^2^LEN Follow-up study

Food group	Food items included
Vegetables	
Leafy vegetables	Lettuce, spinach, chard, fenugreek, wild greens
Fruit vegetables	Capers, tomatoes, aubergine, courgette, sweet peppers, pumpkin, artichoke, okra, mushroom
*Cucurbitacea*	Cucumber, melon, watermelon, bitter melon
*Apiaceae*	Celery, carrot, herbs (coriander, parsley, chervil, dill), parsnip
Other root vegetables	Turnip or swede, radish, beetroot, ginger, taro
Maiz/Corn	Sweet corn
*Alliums*	Onion, garlic, leek
*Brassicaceae*	Brussels sprouts, broccoli, cabbage, cauliflower, coleslaw
Potatoes	Mashed potatoes, baked/roasted/casserole potatoes, chips/french fries, potatoes in salad, potato dumping/bread dumpling/gnocchi, potato tortilla
Pickled vegetables	Cucumber, radish, cabbage
All vegetables	Average intake of all above
Fruits	
Hard fruits	Apple, pear
Citrus fruits	Lemon, orange, mandarin/tangerine, grape-fruit, kiwi
Oily fruits	Olives, avocado
Fruit juice	Freshly squeezed fruits
Berries	Blueberries, strawberries, raspberries (‘*forest berries’*)
Nectarines	Nectarine, apricot, peach
Dried fruits	Raisin, prune
Tropical fruits	Mango, pineapple (banana assessed individually)
Canned fruits	Any canned fruits
Dark pigmented fruit	Cherries, rhubarb, grape, fig, plum
All fruits	Average intake of all above

### Statistical analyses

Sampling probability weights were used to standardise prevalences by gender and age to a European Standard Population.

Multivariable logistic regression was used to assess the relationship between food consumption and CRS within each country, controlling for education, employment, smoking status (never, ex-smoker, current smoker), BMI, age, gender, supplement use and TEI. The country level logistic analyses were weighted to take into account the case–control sampling selection. Negative binomial regression was used to assess the relationship between food consumption and asthma score within each country. This analysis controlled for the same variables and used the same sampling weights as in the logistic regression described above. There was only weak collinearity between the variables when we tested this in each of the multivariable models. The regression coefficients from the country level analyses were meta-analysed to give an overall coefficient. The I^2^ statistic was used to assess heterogeneity between countries. Simes procedure was used to correct statistical estimates derived from multiple testing [18].

All analyses were run using Stata 13.1 (StataCorp, 4905 Lakeway Drive, College Station, Texas 77845 USA).

## Results

The main characteristics of the 3202 participants with valid data on diet and asthma score are summarised in Table 2. Of these, 22.8% reported having at least 1 symptom of asthma (asthma score = 1) whereas 9.3% had 3 or more symptoms. CRS was reported by 23.4% of individuals. Over half of all participants reported eating fruits or vegetables 5 times a week, with Portugal and Poland having the highest intake of these food groups.

**Table 2:** General characteristics of the study population (based on individuals with complete data on dietary exposures and asthma score)

Variables	Countries					
	Denmark	Finland	Sweden	United Kingdom	Germany	The Netherlands
	Odense (359)	Helsinki (160)	Total (1261)	Total (173)	Total (376)	Amsterdam (215)
Age, years; mean (SD)	48.1 (14.5)	46.8 (15.1)	45.7 (15.1)	51.6 (13.2)	48.8 (15.6)	52.6 (13.9)
Males, n (%)	162 (45.1)	62 (38.8)	556 (44.1)	70 (40.5)	152 (403)	111 (51.6)
BMI (kg/m^2^)	27.4 (14.8)	26.5 (4.6)	25.9 (7.2)	27.1 (5.6)	26.3 (4.8)	25.7 (3.7)
Age at completing full-time education; years (SD)	23.4 (5.5)	23.5 (5.5)	24.5 (7.7)	18.1 (3.6)	20.6 (5.2)	20.2 (4.6)
Employment status						
Employed	188 (52.7)	94 (58.9)	737 (58.5)	85 (49.7)	196 (52.0)	103 (47.9)
Retired	82 (23.0)	32 (20.0)	199 (15.8)	39 (22.8)	88 (23.3)	56 (21.1)
Unemployed	11 (3.1)	3 (1.9)	38 (3.0)	4 (2.3)	12 (3.2)	5 (2.3)
Other	76 (21.5)	31 (19.4)	286 (22.7)	43 (25.1)	81 (21.5)	51 (23.7)
Smoking						
Never smokers	155 (43.4)	83 (51.9)	672 (53.3)	77 (44.5)	183 (48.4)	84 (39.1)
Ex-smokers	102 (28.6)	37 (23.1)	428 (33.9)	70 (40.5)	131 (34.7)	88 (40.9)
Current smokers	100 (28.0)	40 (25.0)	162 (12.8)	26 (15.0)	64 (16.9)	43 (20.0)
Asthma score; N (%)						
0	145 (40.4)	96 (59.6)	583 (46.2)	66 (38.2)	161 (42.6)	100 (41.0)
1	85 (23.7)	31 (19.3)	276 (21.9)	37 (21.4)	107 (28.3)	40 (18.6)
2	50 (13.9)	15 (9.3)	195 (15.5)	22 (12.7)	47 (12.4)	37 (17.2)
3	47 (13.1)	10 (6.2)	114 (9.0)	17 (11.5)	35 (9.3)	23 (10.7)
4	24 (6.7)	7 (4.4)	61 (4.8)	26 (15.0)	16 (4.2)	12 (5.6)
5	8 (2.2)	2 (1.2)	33 (2.6)	5 (2.9)	12 (3.2)	3 (1.4)
Chronic rhino-sinusitis; n (%)	63 (17.6)	29 (17.8)	234 (18.3)	22 (12.6)	62 (16.2)	52 (23.9)
Asthma ever (n; %)	115 (32.0)	44 (27.0)	510 (39.8)	80 (45.7)	83 (21.7)	44 (20.2)
CRS only (n; %)	42 (11.7)	17 (10.4)	102 (8.0)	10 (5.7)	38 (9.9)	40 (18.4)
Both asthma ever and CRS (n; %)	21 (5.9)	12 (7.4)	132 (10.3)	12 (6.9)	23 (6.0)	12 (5.5)
Total Energy Intake (TEI)	2577 (761)	3197 (1140)	3110 (978)	2833 (889.6)	2821 (1049)	2817 (827)
Use of nutritional supplements, n (%)	143 (40.4)	70 (43.5)	325 (26.0)	58 (33.7)	102 (27.1)	88 (41.0)
% people eating fruits (all types) ≥5 times/week	202 (56.4)	93 (57.1)	717 (56.0)	101 (57.7)	213 (55.8)	114 (52.3)
% people eating total vegetables (all types) ≥5 times/week	224 (62.4)	128 (78.5)	906 (70.7)	92 (52.6)	194 (50.7)	78 (35.8)

Variables	Countries			
	Portugal	Belgium	Poland	Total
	Coimbra (266)	Ghent (148)	Total (244)	3202
Age, years; mean (SD)	47.1 (15.0)	45.7 (15.1)	49.7 (15.7)	47.6 (15.1)
Males, n (%)	93 (35.0)	71 (48.0)	104 (42.6)	1381 (43.1)
BMI, kg/m^2^ (SD)	25.9 (5.1)	24.9 (4.4)	27.4 (5.2)	26.3 (5.2)
Age at completing full-time education; years (SD)	20.1 (4.6)	20.6 (6.6)	20.4 (3.4)	22.4 (6.6)
Employment status				
Employed	140 (52.6)	75 (51.0)	89 (38.0)	1707 (53.6)
Retired	56 (26.5)	26 (17.7)	86 (36.8)	664 (20.8)
Unemployed	11 (4.1)	3 (2.0)	12 (5.1)	99 (3.1)
Other	59 (22.2)	30 (22.4)	47 (20.0)	717 (22.5)
Smoking				
Never smokers	172 (64.7)	75 (50.7)	111 (45.7)	1612 (50.4)
Ex-smokers	56 (21.1)	45 (30.4)	78 (32.1)	1035 (32.2)
Current smokers	38 (14.3)	28 (18.9)	54 (22.2)	555 (17.3)
Asthma score				
0	109 (41.0)	57 (38.5)	78 (32.0)	1395 (43.5)
1	49 (18.4)	34 (23.0)	73 (29.9)	732 (22.8)
2	41 (15.4)	22 (14.9)	34 (13.9)	463 (14.4)
3	27 (910.2)	17 (11.5)	28 (11.5)	318 (9.9)
4	23 (8.7)	12 (8.1)	17 (7.0)	198 (6.2)
5	17 (6.4)	6 (4.1)	14 (5.7)	100 (3.1)
Chronic rhino-sinusitis; n (%)	78 (29.2)	43 (29.1)	50 (20.2)	633 (19.5)
Asthma ever (n; %)	59 (22.1)	23 (15.5)	37 (15.0)	995 (30.7)
CRS only (n; %)	44 (16.5)	28 (18.9)	39 (15.8)	360 (11.1)
Both asthma ever and CRS (n; %)	34 (12.7)	15 (10.1)	11 (4.5)	272 (8.4)
Total Energy Intake (TEI); mean (SD)	3195 (1296)	2937 (885)	3211 (1661)	2993 (1072)
Use of nutritional supplements, n (%)	16 (6.0)	50 (33.8)	53 (22.0)	905 (28.4)
% people eating fruits (all types) ≥5 times/week	189 (70.8)	80 (54.1)	158 (64.0)	1867 (57.6)
% people eating total vegetables (all types) ≥5 times/week	206 (77.2)	77 (52.0)	182 (73.7)	2087 (64.4)

**Fig. 1:**
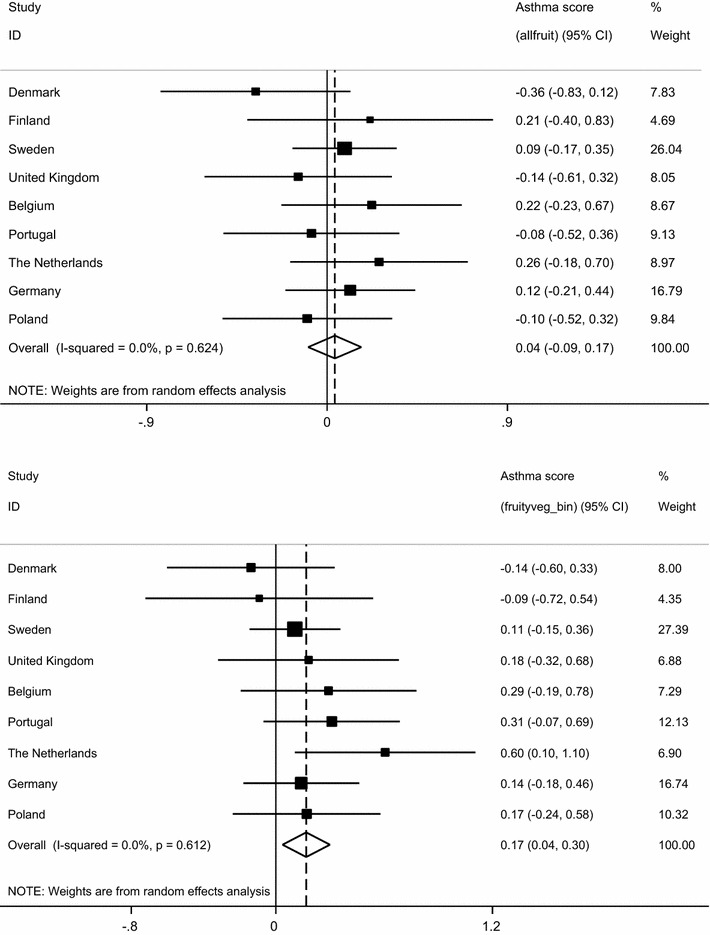
Weighted adjusted negative binomial regressions of asthma score association with total intake of fruits (*top*) and fruity vegetables (*below*) (per centre, and meta-analysis of pooled results)

 The association between asthma score and fruit and vegetable intake is illustrated in Table 3. After controlling for potential confounders, a statistically significant negative association was observed between having an increasing asthma score and eating dried fruits (β-coefficient −2.34; 95% CI −4.09, −0.59; P value = 0.009). No other fruit groups were associated with asthma. Intake of fruity vegetables (which included capers, tomatoes, aubergine, courgette, sweet peppers, pumpkin, artichoke, okra, and mushroom) was positively associated with asthma score (β-coefficient 0.17; 95% CI 0.04, 0.30). Similarly, a higher asthma score was related to intake of alliums vegetables (onion, garlic, leek) (β-coefficient 0.23; 95% CI 0.06, 0.40). Figure 1 illustrates the per-country associations between asthma score and total fruit intake and fruity vegetables. There was no heterogeneity across countries (I^2^ = 0%).

**Table 3:** Association between severity of asthma (asthma score) and fruit and vegetable intake in adults from GA^2^LEN

Fruit and vegetable groups	Asthma score Effect size (β-coefficient (95% confidence intervals)	
	Unadjusted (n = 3206)	Adjusted (n = 2945)
Fruits		
Hard fruits	0.01 (−0.11, 0.14) n = 3196	−0.02 (0.15, 0.11) n = 2940
Bananas	0.03 (−0.14, 0.21) n = 3187	0.04 (−0.19, 0.27) n = 2934
Citrus fruits	−0.05 (−0.19, 0.09) n = 3196	−0.03 (−0.18, 0.12) n = 2938
Oily fruits	0.25 (0.02, 0.48) n = 3196	0.24 (0.01, 0.46) n = 2942
Freshly squeezed fruit	0.16 (−0.03, 0.36) n = 3184	0.18 (−0.01, 0.38) n = 2930
Berries	−0.07 (−0.32, 0.19) n = 3159	−0.12 (−0.37, 0.13) n = 2907
Nectarines	0.26 (−0.10, 0.62) n = 3197	0.16 (−0.33, 0.65) n = 2942
Dried fruits	−*1.89 (*−*3.36,* −*0.42) n* = *3190*	−*2.34 (*−*4.09,* −*0.59) n* = *2937*
Tropical fruits	0.13 (−0.31, 0.56) n = 3194	0.21 (−0.15, 0.55) n = 2940
Canned fruits	−4.62 (−6.50, −2.74) n = 3181	−5.66 (−11.4, 0.07) n = 2930
Dark pigmented fruits	−0.11 (−0.41, 0.19) n = 3201	−0.09 (−0.37, 0.19) n = 2944
All fruits	−0.03 (−0.16, 0.10) n = 3203	0.04 (−0.09, 0.17) n = 2944
Nuts	0.21 (−0.12, 0.54) n = 3192	0.20 (−0.21, 0.61) n = 2935
Vegetables		
Leafy vegetables	0.11 (−0.04, 0.26) n = 3195	0.03 (−0.15, 0.22) n = 2937
Fruity vegetables	*0.16 (0.04, 0.28) n* = *3202*	*0.17 (0.04, 0.30) n* = *2942*
*Cucurbitacea*	0.07 (−0.10, 0.24) n = 3202	−0.02 (−0.22, 0.18) n = 2943
*Apiaceae*	0.05 (−0.12, 0.21) n = 3204	0.05 (−0.09, 0.19) n = 2943
Other root vegetables	0.13 (−0.08, 0.33) n = 3200	0.12 (−0.13, 0.37) n = 2942
Maize/corn	0.41 (−0.12, 0.93) n = 3189	0.47 (−0.04, 0.98) n = 2936
*Alliums*	*0.27 (0.15, 0.39) n* = *3203*	*0.23 (0.06, 0.40) n* = *2944*
*Brassicaceae*	0.30 (0.01. 0.59) n = 3202	0.20 (−0.02, 0.41) n = 2943
Potatoes	0.09 (−0.21, 0.38) n = 3194	0.002 (−0.24, 0.24) n = 2937
Pickled vegetables	−2.32 (−4.17, −0.47) n = 3175	−1.90 (−3.94, 0.14) n = 2924
Legumes	−*2.10 (*−*3.65,* −*0.45) n* = *3196*	−1.98 (−4.13, 0.18) n = 2939
All vegetables	0.12 (−0.001, 0.25) n = 3206	0.11 (−0.03, 0.25) n = 2945

Italics indicate a statistically significant effect size

**Fig. 2:**
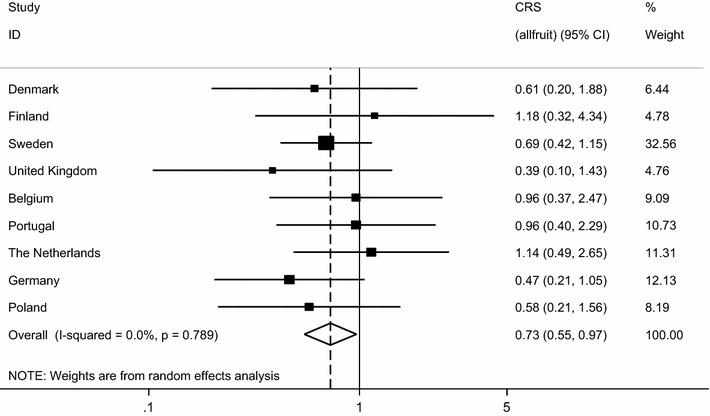
Weighted multivariable analyses of association between CRS with total intake of fruits (per centre, and meta-analysis of pooled results)

 Table 4 shows the associations found between CRS and fruit and vegetable intake. A 27% lower risk of disease was observed in those with a total intake of fruit ≥5 versus those who ate fruit below this cut-off point (OR 0.23; 95% CI 0.55, 0.97). As illustrated in Fig. 2, there was no evidence of heterogeneity between the estimates across countries (I^2^ = 0.0%; P value = 0.62).

**Table 4:** Association between CRS and fruit and vegetable intake in adults from GA^2^LEN

Fruit and vegetable groups	Effect size (odds ratio (95% confidence intervals)	
	Unadjusted (n = 3242)	Adjusted (2970)
Fruit group		
Hard fruit	0.83 (0.64–1.06) n = 3232	0.82 (0.62–1.09) n = 2965
Bananas	1.04 (0.78–1.40) n = 3223	0.99 (0.68–1.44) n = 2959
Citrus fruit	0.78 (0.48–1.26) n = 3232	0.87 (0.52–1.46) n = 2963
Oily fruits	1.40 (0.91–2.16) n = 3232	1.67 (0.91–3.06) n = 2967
Freshly squeezed fruit	0.73 (0.44–1.20) n = 3219	0.74 (0.44–1.24) n = 2954
Berries	1.08 (0.61–1.94) n = n = 3195	1.23 (0.55–2.76) n = 2932
Nectarines	1.42 (0.84–2.41) n = 3233	1.57 (0.79–3.11) n = 2967
Dried fruits	0.95 (0.42–2.14) n = 3226	0.98 (0.42–2.32) n = 2962
Tropical fruits	2.14 (1.10–4.16) n = 3230	2.50 (0.91–6.92) n = 2965
Canned fruits^a^	–	–
Dark pigmented fruits	1.01 (0.71–1.45) n = 3237	1.11 (0.75–1.64) n = 2969
All fruits	*0.75 (0.58*–*0.96) n* = *3239*	*0.73 (0.55*–*0.97) n* = *2969*
Nuts	0.47 (0.21–1.06) n = 3227	0.64 (0.23–1.80) n = 2960
Vegetables		
Leafy vegetables	1.15 (0.86–1.53) n = 3229	1.22 (0.86–1.71) n = 2961
Fruity vegetables	1.16 (0.87–1.53) n = 3237	1.22 (0.81–1.85) n = 2967
*Cucurbitacea*	1.15 (0.85–1.56) n = 3238	1.03 (0.73–1.44) n = 2968
*Apiaceae*	1.22 (0.93–1.62) n = 3239	1.22 (0.90–1.64) n = 2968
Other root vegetables	1.63 (0.98–2.70) n = 3235	1.77 (0.89–3.53) n = 2967
Maize/corn	1.64 (0.55–4.87) n = 3224	1.74 (0.42–7.22) n = 2961
*Alliums*	1.19 (0.91–1.55) n = 3238	0.99 (0.68–1.42) n = 2969
*Brassicaceae*	1.09 (0.73–1.62) n = 3237	1.05 (0.67–1.65) n = 2968
Potatoes	*2.27 (1.47–3.52) n* *=* *3229*	*1.82 (1.03–3.23) n* *=* *2962*
Pickled vegetables	1.73 (0.88–3.4) n = 3210	1.61 (0.72–3.59) n = 2949
All vegetables	1.11 (0.80–1.54) n = 3242	1.09 (0.67–1.77) n = 2970
Legumes	1.54 (0.51–4.64) n = 3231	1.24 (0.30–5.10) n = 2964

Italics indicate a statistically significant effect size

^a^Not enough people with data on this exposure to carry out analyses

After applying Simes procedure, the statistical significance of the association between asthma score and dried fruits was attenuated (P value = 0.05), and all the other associations were no longer statistically significant (>0.15).

## Discussion

In this multi-national study of adults participating in the GA^2^LEN Follow-up survey, asthma symptom score and CRS were negatively associated with dietary intake of dried fruits and total fruit intake, respectively. Asthma symptom score was also positively associated with a higher intake of fruity vegetables and alliums. These associations were observed after adjusting for several potential confounders, which included socio-economic, smoking, and lifestyle-related variables (including BMI, TEI, and nutritional supplement use). After controlling for multiple comparisons, the statistical significance of these associations was lost.

To our knowledge, this is the first multi-national population-based study to examine the association between asthma, CRS and allergic rhinitis, with fruit and vegetable intake, using a standardised method to ascertain both respiratory outcomes and dietary exposures. The results of this study were weighted to make results generalizable to the European adult population. We used an asthma score to ascertain individuals with a variety of symptoms, for its good predictability to ascertain outcomes related to asthma [14, 19]. Asthma is characterised for its clinical phenotypic heterogeneity and temporal phenotypic variability. Being a multi-categorical measure, the score provides more power to detect risk factors for asthma [19].

The GA^2^LEN FFQ was translated into each of the participant countries’ languages following international guidelines, and was previously piloted and validated in a subsample of 5 participating countries [11]. The FFQ uses a semi-quantitative approach to enquiring about the frequency of intake of 250 food items, which includes staple foods representative of each nation, but also foods that are commonly consumed in all these countries. The GA^2^LEN FFQ is being used in several other multi-national countries and appears to be a functional and accurate tool to ascertain usual dietary intake [15]. Given the large number of dietary exposure studied, we used Simes procedure to adjust the P values for multiple testing. This method has more power to identify true associations and its use is helpful when there are several highly correlated variables, as it is the case of dietary exposures [18].

The absence of robust evidence suggesting an association between dietary intake of fruits and vegetables with respiratory outcomes in this study has been confirmed in other population-based observational studies. Several authors have reported no association between asthma risk and intake of citrus fruits. As reported in other studies, we did not observe an association between the outcomes studies and citrus fruits [3, 20–22] nor with vitamin C, for which observational studies show mixed evidence of a beneficial effect [23].

We did find a negative association between dried fruit intake and asthma score, which remained statistically significant after controlling for multiple comparisons. Recent experimental evidence has demonstrated in an asthma-induced model in rats, that administering *V. vinifera* dried fruits inhibited the recruitment of inflammatory cytokines (IL)-4, IL-5, IL-1β, tumour necrosis factor, as well as IgE levels, and circulating levels of eosinophils in blood/serum and broncho-alveolar fluid [24]. Treatment with raisin extract also normalised lung function and histamine levels compared to control animals. Although no experimental evidence has demonstrated that prunes might exert similar effects, it has been proposed that the potential beneficial role of prunes on asthma might be mediated through their role in maintaining the gut microbiota balance [25]. Our findings of a negative association between dried fruits (raisins and prunes) might be explained at least partly by these biological mechanisms.

Several other studies have used a more integrative approach to elucidate the association between asthma and dietary exposures using dietary patterns, derived from Principal Component or Factor analysis, or through other indexes. However, dietary patterns that include fruits and vegetables as main food contributors have so far been unrelated to prevalence [9] or risk of adult asthma [26]. The uniformity of the associations observed per country in our study, and the absence of heterogeneity observed in most analyses, would lend further support to the notion that in general intakes of fruits and vegetables are not strongly associated with adult asthma.

Fruits and vegetables are also rich in various subclasses of flavonoids, for which strong anti-oxidant, anti-inflammatory and anti-allergic properties have been demonstrated in experimental studies of induced asthma [27]. These results have been echoed in some observational studies in adults showing a reduced risk of BHR [7] or asthma incidence [28], though others have reported no association with current asthma or allergic symptoms [29]. This is partly explained by the differences in the subclasses studied. In our study, we found some evidence that a lower risk of CRS was associated with a higher intake of fruits, which could partly be explained by the high content of vitamin C and flavonoids in them. We err on the cautious side though as this association was no longer statistically significant after controlling for multiple testing.

Due to the cross-sectional nature of our analysis, we cannot ascribe causality (or lack of) in the association between asthma, CRS, and allergic rhinitis with dietary intake of fruits and vegetables. Although we adjusted for several important potential confounders, there are likely to be other unmeasured confounders involved in the complex association between asthma and diet.

In conclusion, we found no consistent evidence for an association of asthma and allergic rhino-sinusitis with fruit and vegetable intake. The overall effect size observed for CRS and total fruit intake is suggestive of a protective effect, but this needs to be taken with caution given the multiple comparisons carried out in the study.

## Abbreviations

GA^2^LEN: The Global Asthma and Allergy Network of Excellence
FFQ: food frequency questionnaire
CRS: chronic rhino-sinusitis
TEI: total energy intake
BMI: body mass index
